# The use of research questionnaires with hearing impaired adults: online vs. paper-and-pencil administration

**DOI:** 10.1186/1472-6815-12-12

**Published:** 2012-10-29

**Authors:** Elisabet Sundewall Thorén, Gerhard Andersson, Thomas Lunner

**Affiliations:** 1 Eriksholm Research Centre, Oticon A/S, Snekkersten, Denmark; 2 Department of Clinical and Experimental Medicine, Division of Technical Audiology, Linköping University, Linköping, Sweden; 3 The Swedish Institute for Disability Research, Örebro and Linköping University, Linköping, Sweden; 4 Department of Behavioural Sciences and Learning, Linköping University, Linköping, Sweden; 5 Department of Clinical Neuroscience, Karolinska Institutet, Stockholm, Sweden

## Abstract

**Background:**

When evaluating hearing rehabilitation, it is reasonable to use self-report questionnaires as outcome measure. Questionnaires used in audiological research are developed and validated for the paper-and-pencil format. As computer and Internet use is increasing, standardized questionnaires used in the audiological context should be evaluated to determine the viability of the online administration format.

The aim of this study was to compare administration of questionnaires online versus paper- and pencil of four standardised questionnaires used in hearing research and clinic. We included the Hearing Handicap Inventory for the Elderly (HHIE), the International Outcome Inventory for Hearing Aids (IOI-HA), Satisfaction with Amplification in Daily Life (SADL), and the Hospital Anxiety and Depression Scale (HADS).

**Methods:**

A cross-over design was used by randomly letting the participants complete the questionnaires either online or on paper. After 3 weeks the participants filled out the same questionnaires again but in the other format. A total of *65* hearing-aid users were recruited from a hearing clinic to participate on a voluntary basis and of these *53* completed both versions of the questionnaires.

**Results:**

A significant main effect of format was found on the HHIE (*p* < 0.001), with participants reporting higher scores on the online format than in the paper format. There was no interaction effect. For the other questionnaires were no significant main or interaction effects of format. Significant correlations between the two ways of presenting the measures was found for all questionnaires (p<0.05). The results from reliability tests showed Cronbachs α’s above .70 for all four questionnaires and differences in Cronbachs α between administration formats were negligible.

**Conclusions:**

For three of the four included questionnaires the participants’ scores remained consistent across administrations and formats. For the fourth included questionnaire (HHIE) a significant difference of format with a small effect size was found. The relevance of the difference in scores between the formats depends on which context the questionnaire is used in. On balance, it is recommended that the administration format remain stable across assessment points.

## Background

When evaluating hearing rehabilitation, it is reasonable to use self-report questionnaires as outcome measures to elicit the patient’s opinion of specific hearing rehabilitation efforts and hearing aid use in daily real-life situations [1]. However, as pointed out by Saunders [2], the process of completing questionnaires can be very time consuming and cumbersome compared with performing basic objective tests in a clinical setting. Some of the disadvantages involved when delivering questionnaires in the paper-and-pencil format could be overcome by using an online format that is accessible from the patient’s own home. Advantages of using an online format include the ease of completion, ease of data handling and lowered risk of data entry errors during transcription [3,4]. Additionally, online questionnaires may also lower the costs of a survey and increase the response rate, thereby having a positive effect on survey validity [5]. Moreover, some patients prefer to complete questionnaires using a computer rather than using paper and a pencil. For instance, this may occur with patients who feel uncomfortable when completing a specific questionnaire in front of the person who has been responsible for their rehabilitation [6,7]. However, there are also disadvantages to using online questionnaires. For example, all questionnaires used in audiological research are developed and validated for the paper-and-pencil format. There can be significant differences between ways of administering questionnaires that may affect the quality and characteristics of the data [8]. Because of these potential disadvantages, the American Psychological Association has suggested that norms and criteria for online questionnaires should be obtained before the questionnaires can be used as a replacement for the paper-and-pencil questionnaires [9,10]. Furthermore Saunders [2] argued that computers are impersonal, that people have negative attitudes towards computers and that many people are still unfamiliar with using this type of technology. This technological unfamiliarity is worth considering, especially for older people who may be both less familiar with computers than younger people and more likely to be hearing impaired.

As computer and Internet use is increasing, especially among the elderly [11], standardized questionnaires used in the audiological context should be evaluated to determine the viability of the online administration format. It is likely that at least parts of future hearing rehabilitation will take place online, necessitating an examination of the psychometric properties and of test characteristics of online administered questionnaires [12-14].

The aim of this study was to compare online versus paper-and-pencil administration of four questionnaires measuring hearing-related issues. The questionnaires measure hearing-aid benefit, hearing-aid satisfaction, activity limitation and participation restriction. The chosen questionnaires are considered reliable and internally valid [15,16], but none have been validated for Internet use. In a randomised cross-over design, half of the participants answered the online versions of the questionnaires first and the paper versions second, and the other half of the participants answered the questionnaires in the opposite order.

## Method

### Recruitment and procedure

Hearing impaired adults who had finished their hearing aid rehabilitation at the University hospital of Linköping were invited to participate in the study. In addition to having completed hearing aid rehabilitation, the following inclusion criteria were defined: 1) over the age of 18 years, 2) able to access the Internet, 3) regular user of e-mail and 4) fluent in Swedish. No participants were excluded from the study because of level of hearing loss or hearing-aid use. The participants were randomised to first complete the questionnaires by paper-and-pencil or via the Internet. After three weeks, the participants were retested with the alternate method of administration. The test-retest interval of three weeks was considered short enough to minimize clinical change and yet long enough to reduce recall bias.

The participants were informed about the study through information letters. They were informed that participation in the study was voluntary, that they could choose to abandon the study at any time without providing an explanation, and that leaving the study before completion would not affect their further treatment at the hearing clinic. Before they were included in the study, the participants were asked in a separate questionnaire about their Internet access, e-mail usage and educational level.

### Questionnaires

The first questionnaire used in the study was the 25-item Hearing Handicap Inventory for the Elderly (HHIE) [17,18]. For each of the 25 items, there are three potential responses: *yes* (4 points), *sometimes* (2) or *no* (0). A higher score corresponds to being more affected by hearing loss in terms of greater perceived activity limitation and greater participation restriction.

The second questionnaire used was the International Outcome Inventory for Hearing Aids (IOI-HA), which is a seven-item questionnaire that measures the benefit of hearing aids [19-21]. Each item focus on a different topic: 1) daily use, 2) benefit, 3) residual activity limitations, 4) satisfaction, 5) residual participation restriction, 6) impact on others and 7) quality of life. Each item has five potential responses ranging from the worst to the best outcome. A higher score on this questionnaire indicates a better outcome of hearing aid use.

The third questionnaire was the Satisfaction with Amplification in Daily Life (SADL) [22], which is a 15-item questionnaire that measures the benefits and positive effects of hearing aids on a seven-point scale. Higher scores in the SADL questionnaire indicate higher satisfaction and more benefit from hearing aid use.

The final questionnaire used was the 14-item Hospital Anxiety and Depression Scale (HADS) [23]. This questionnaire measures symptoms of anxiety and depression during the week prior to participation. Each item has four possible responses (score 0–3). A higher score on this questionnaire indicates more symptoms of anxiety and depression.

### Paper questionnaires

The paper format questionnaires contained written instructions for completing the surveys and a prepaid return envelope. All items were mailed to the participants at their home address. The questionnaires were placed in the following order: HHIE, IOI-HA, SADL and HADS. However, the participants were not instructed to fill out the questionnaires in a specific order.

### Web questionnaires

The Customer Relationship Management (CRM) system Communigator (version 5) was used to generate individualized e-mails for all participants. Each e-mail contained a direct link to the online survey. The individualized e-mail served as the personal login to the survey, thus controlling access to the online survey. Subjects could only fill out the survey once. The responses to the questionnaires were automatically stored in a database. The questionnaires were presented in the following order: HHIE, IOI-HA, SADL and HADS. In total, sixty-five questions were presented, the same as were presented via the paper version. It took approximately 25–30 minutes for participants to complete all questions.

If participants did not respond to either survey form within a two-week timeframe, they were called by telephone to check whether the e-mail or letter had been received.

### Participants

Participants consisted of 65 hearing-aid users from the hearing aid clinic who volunteered for the study. A majority of the included participants were men (80%), and the ages ranged from 36 to 90 years (M= 68.3; SD 11.3 years). A third (32%) of the participants had an education equivalent to university level (see Table 1 for more descriptive information).

**Table 1:** Descriptive data of the participants

	**Volunteered participants**	**Completers of both formats**
**N**	65	53
**F:M**	13:52	13:40
**Mean Age (range)**	68.3 (36–90)	67.8 (36–90)
**University degree (%)**	32	30

The participants were randomised by an independent person not involved in the study to participate in either group 1 (*n* = 32) or in group 2 (*n* = 33). Group 1 consisted of 26 men (81%) and 6 women (19%). The first task for participants in Group 1 was to fill out the questionnaires by paper and pencil, and after three weeks, to complete the same questionnaires online. Group 2 consisted of 26 men (79%) and 7 women (21%) and completed the questionnaires in the opposite order, resulting in a cross-over design. Participants reported e-mail problems in a few cases, and in these particular cases, a new e-mail was sent.

The medical ethics committee in Linköping, Sweden, approved the protocol of this study.

### Statistical analysis

Significance testing of differences in the questionnaire administration format (paper-& pencil and Internet) and order (Paper first or Internet first) was determined using 2x2 mixed ANOVAs with format of the questionnaire as the repeating factor. Analyses of the data were conducted using the software package STATISTICA (version 10). After ANOVAs were performed calculation of effect size (Cohen’s d) was used. According to Cohen [24], d = 0.2-0.5 is small; d = 0.5-0.8 is medium and d > 0.8 is considered to be a large effect. Pearson’s product–moment correlation and an internal consistency test (Cronbach’s α) were used to further analyse the data.

## Results

Of the 65 included participants, 53 completed the questionnaires both on paper and on the Internet. Three participants withdrew from the Internet part of the study because of technical problems related to either Internet access or to other computer problems. Another nine participants did not complete either version of the questionnaires and did not provide a reason for declining to participate.

Initial analyses of the data showed that the results from IOI-HA and SADL questionnaires had a Normal distribution and that the results from HHIE and HADS were skewed. Before further analyses were performed, the data from HHIE and HADS underwent square root transformation. This transformation redistributed the data to a Normal distribution and allowed the inclusion of the data in further analyses (see Table 2 for summary of the raw data).

**Table 2:** Mean (SD), Median and 25-75% range for the questionnaires in the different formats

	**Paper**		**Internet**	
	**Mean (SD)**	**Median (25%-75%)**	**Mean (SD)**	**Median (25%-75%)**
**HHIE**	26.0 (16.5)	22.0 (14.0-34.0)	29.9 (17.3)	24.0 (18.0-40.0)
**IOI-HA**	26.3 (4.7)	26.0 (23.0-30.0)	26.5 (5.2)	27.0 (23.0-31.0)
**SADL**	4.8 (0.7)	4.8 (4.3-5.3)	4.6 (0.6)	4.6 (4.3-5.1)
**HADS**	7.3 (5.9)	6.0 (3.0-10.0)	6.6 (5.4)	5.0 (3.0-8.0)

Data from HHIE and HADS are not normally distributed. N = 53.

### Effects of order and format of administration

Possible order effects and format effects were examined using a 2-way ANOVA with questionnaire format as the repeating factor (see Table 3).

**Table 3:** **Result (F-values) from ANOVA. Data for HHIE and HADS are transformed,** ***N = 53***

	**Main effect**		**Interaction**
	**Format**	**Group**	
**HHIE**	11.72***	0.68	0.37
**IOI-HA**	0.26	1.31	2.14
**SADL**	1.32	1.81	1.75
**HADS**	0.18	3.70	1.15

Data for HHIE and HADS are transformed (square root) to become Normal distributed.

***p < .001.

The results showed a significant main effect of format for the HHIE questionnaire (F_1,51_=11.72; p < 0.001). Participants indicated on average a higher score of 3.9 points on the HHIE in the online version of the questionnaire than in the paper version. The effect size of the result was small (d = 0.37). Other than this, no significant main effect of group or interaction effects were observed for HHIE.

In the remaining questionnaires (IOI-HA, SADL and HADS), no significant main effects of group or format were observed. Further, the results showed no significant interaction effect of group and format. The lack of interaction effect indicates that the order in which the participants completed the questionnaires did not matter.

### Correspondence between the two formats

Pearson’s product–moment correlation was used to analyse the correspondence between the online and paper formats. Significant correlations were found for all four questionnaires (p < .001), (see Table 4). The strongest correlation was observed for the total score from HHIE (*r* = .86, p < 0.001). The weakest correlation was observed for the total score from SADL (*r* = .56, p < 0.001).

**Table 4:** **Results from internal consistency (Cronbach’s α) and results from correlation between the formats (Pearson), *N = 53***

	**Cronbach’s α**		**Paper x Internet**
	**Paper**	**Internet**	**Correlation**^**a**^
**HHIE**	0.92	0.92	0.86
**IOI-HA**	0.82	0.82	0.84
**SADL**	0.76	0.75	0.56
**HADS**	0.84	0.85	0.67

^a^All correlations are significant at the *P* = .001 level.

Additionally, the reliability of each questionnaire was tested by comparing the internal consistency (Cronbach’s α) for the Internet and paper formats (see Table 4). The Cronbach’s α result was above .70 for all of the four questionnaires, and the differences between formats were negligible for each questionnaire. For HHIE and IOI-HA, no differences between the Internet and paper versions were measured using Cronbach’s α. For both SADL and HADS, a small difference of .01 was observed between the Internet and paper versions.

## Discussion

This study was designed to evaluate the reliability across formats of four different questionnaires that are commonly used in hearing aid rehabilitation and research. Hearing-aid users are commonly old as a group and are likely to use computers and the Internet to a lesser extent than younger age groups, for whom Internet administration of questionnaires has been validated previously e.g. [25]. The present study indicates that at least a proportion of hearing-aid users is willing and has the capacity to use the Internet when completing questionnaires. It is worth noting that the invitation letters were sent out to an equal number of men and women, but a majority of the responses were from men (80%), which may reflect more willingness to use the Internet among men in this age group.

Our results showed that there were no order effects for format presentation. The lack of order effects indicates that it does not matter whether the participants filled out the online or the paper versions of the questionnaires first.

The results of the current study are consistent with previous studies with other target groups [26,27] in which comparisons have been made between the two administration formats. Scores on the outcome measures showed no significant differences across questionnaire formats for three out of the four questionnaires administered in the current study. The psychometric results for the HHIE questionnaire are consistent with what has been reported in previous studies when using the HHIE in the paper format [15,28]. A significant main effect of format showed that the participants in general rated a higher HHIE score of 3.9 points, on a scale of 100 points, in the Internet format than in the paper format. Earlier studies have hinted that participants may reveal more about themselves when communicating via a computer [29]. This might explain the higher degree of hearing difficulties reported by participants using the online format compared with the paper format. The effect size of the difference between the formats was small therefore it depends on the context where the questionnaire will be used in, which the actual relevance of a difference of this magnitude is [30]. Buchanan’s suggestion [10] that separate norms should be derived for Internet-based and paper-based questionnaires may therefore be relevant for hearing-related measures as well, and important when it comes to the HHIE questionnaire.

Internal consistencies as evaluated using Cronbach’s α were well above .70 for all questionnaires (.75–.92). According to earlier research, this indicates that the internal consistency and reliability for each questionnaire across formats is good (IOI-HA, SADL & HADS) or excellent (HHIE) [31]. The Cronbach’s α values from the online versions of the questionnaires were in line with results from earlier studies with the paper-and-pencil format, all of which presented the questionnaires to a similar population and using the same language as the current study [15,17,22,27,32,33].

Pearson’s product–moment correlation results of 0.74 and above indicate high reliability across the two forms of administration [31]. The results from the questionnaires in this study are well within the acceptable range for validity tests of this nature [34]. The correlations for two of the questionnaires (HHIE and IOI-HA) were above 0.74, and the correlations for the remaining two questionnaires (SADL and HADS) were below 0.74 but still significant. The relatively small number of test subjects could account for the somewhat lesser correlations obtained from the SADL and HADS surveys. The 3-week interval between questionnaire administrations could have lowered the correlations for the HADS questionnaire because separating the administration dates required participants to report their moods for two different weeks [7]. The interval between test 1 and 2 was determined by the experimenters to be short enough to exclude clinical change but long enough to reduce recall bias. It is likely that interval length affected questionnaire results, and follow-up studies should examine other interval options.

### Limitations

In this study, the total score for each questionnaire was analysed. For three of the four used questionnaires (HHIE, SADL and HADS), the questions can be divided into sub-categories to analyse the differences according to administration format, but that analysis was not performed because of the low power in this study. The conclusions drawn in this study are therefore based on the total score for each questionnaire and not on any subscales.

The participants were all clinical patients recruited via the public health care system, which suggests that the group is representative for the hearing aid population. However, possession of an e-mail account was required for participation in the study. This requirement limits generalisability because the results from this experiment can only be generalised to hearing impaired adults having some type of regular Internet activity, even though they are clinically representative. Including more female participants in the present study would have improved the generalisability of the conclusions. An equal number of men and women were invited to participate in the study, but more men than women responded, indicating that men may be more willing than women to participate in computer-related research studies.

## Conclusions

In conclusion, there is sufficient evidence that participants’ scores remained consistent across administrations and formats for three of the four included questionnaires. For the fourth included questionnaire (HHIE) a significant difference of format with a small effect size was found. The relevance of the difference in scores between the formats depends on which context the questionnaire is used in. On balance, it is recommended that the administration format remain stable across surveys, as indicated in related research [25]. The current findings support claims made in earlier studies [4,25-27], particularly, the claim that online questionnaires provide the advantages of saved resources and more complete answers when compared with paper-and-pencil questionnaires.

## Competing interests

The authors declare that they have no competing interests.

## Authors’ contributions

This work is performed in close collaboration by the authors. EST initiated and designed the study together with GA and TL. EST coordinated the study and was responsible for the distribution and collection of the questionnaires. EST analysed the data and drafted the manuscript in close collaboration with GA and TL. All authors read and approved the final manuscript.

## Pre-publication history

The pre-publication history for this paper can be accessed here:

http://www.biomedcentral.com/1472-6815/12/12/prepub
